# ﻿Comparison of muscle structure and transcriptome analysis of eyed-side muscle and blind-side muscle in *Cynoglossussemilaevis* (Osteichthyes, Cynoglossidae)

**DOI:** 10.3897/zookeys.1230.139837

**Published:** 2025-03-06

**Authors:** Zhenming Lü, Yuzhen Wang, Jing Yu, Yijing Yang, An Xu, Li Gong, Jing Liu, Fenghui Li, Liqin Liu

**Affiliations:** 1 National Engineering Laboratory of Marine Germplasm Resources Exploration and Utilization, College of Marine Sciences and Technology, Zhejiang Ocean University, Zhoushan 316022, China Zhejiang Ocean University Zhoushan China; 2 National Engineering Research Center for Facilitated Marine Aquaculture, Zhejiang Ocean University, Zhoushan 316022, China Zhejiang Ocean University Zhoushan China

**Keywords:** *Cynoglossussemilaevis*, differentially expressed genes, flatfishes, gluconeogenesis, glycolysis, muscle development, regulatory mechanism, transcriptome analysis

## Abstract

*Cynoglossussemilaevis* (Osteichthyes, Cynoglossidae) is one of the most significant commercial marine fish species in China and has evolved a specialized asymmetrical body axis. In addition, *C.semilaevis* displays different muscle thickness between the eyed side and the blind side. However, the mechanisms underlying the muscle development difference between the two sides in *C.semilaevis* are unclear. In this study, we generated the first comparative investigation on the structure of muscle cells, and transcriptome analysis between the eyed-side muscle (ESM) and blind-side muscle (BSM) in *C.semilaevis*. Histological assays showed the obvious mosaic appearance of muscles on both the eyed side and blind side. However, the number of new muscle cells in ESM was significantly more than that in the BSM group. Comparative analyses of RNA-seq data showed that 1177 differentially expressed genes (DEGs) were identified between ESM and BSM groups, including 291 up-regulated and 886 down-regulated genes. The expression levels of myosin family genes (actin, myosin-binding protein C, titin, troponin, tnnil, and astrotactin-2) were significantly higher in ESM and might be a candidate regulator of muscle filament assembly in *C.semilaevis*. Murine double minute 2 (Mdm2) and cyclin A2 (ccna2) were also up-regulated in ESM, which indicates that the muscle development difference between ESM and BSM in *C.semilaevis* might be owing to the variation in myofibroblast proliferation. In addition, KEGG pathway enrichment analyses suggested that the glycolysis/gluconeogenesis pathway may be involved in the muscle development of *C.semilaevis*. Taken together, this study may provide useful information to understand the molecular mechanism of muscle development in flatfishes.

## ﻿Introduction

External asymmetry is common in flatfishes, though rare in vertebrates. However, the asymmetry of flatfishes is astonishing because the flatfish larvae are perfectly symmetrical (Janvier 2008). The transformation of symmetrical larvae into asymmetrical juvenile is completed by the process of metamorphosis, in which the entire head structure is rapidly remodeled, involving the migration of one eye to the opposite side of the head (Schreiber 2006). After metamorphosis, the adult flatfish have a pigmented ‘eyed’ (dorsal) side and a non-pigmented ‘blind’ (ventral) side. In addition to eye-sidedness, another characteristic of postembryonic asymmetry is the presence of a much larger muscle thickness on the eyed side of adult individuals compared to the blind side in flatfishes (Hagen et al. 2008; Lü et al. 2021). In fish, muscle accounts for approximately 50%-70% of the body weight, and is the main consumed product (Valente et al. 2013). Its development influenced fish meat production and aquaculture development. Therefore, understanding the underlying mechanisms controlling fish muscle development of flatfishes is not only crucial to the aquaculture industry, but also provides insights for understanding the molecular mechanism of the asymmetrical body axis.

Muscle development in fish is different from that of mammals in which hypertrophic and hyperplasic growth continues throughout much of the life cycle (Johnston 1999; Veggetti et al. 1999). It is a polygenic and complex biological process that is influenced by a series of genes and signaling pathways (Ahammad et al. 2019; Rescan 2019; Chen et al. 2023; Yao et al. 2024). There have been many studies investigating how these genes and signaling pathways affect muscle development and growth in fish. For example, genes in the growth hormone-insulin-like growth factor-I (GH/IGF) axis have been identified as key genes in regulating muscle growth and development in Atlantic salmon (*Salmosalar*) (Johnston et al. 2003), and the glycolysis/gluconeogenesis pathway could influence the growth of starry flounder (*Platichthysstellatus*) and *C.semilaevis* (Osteichthyes, Cynoglossidae) (Wang et al. 2021). Zhang et al. (2021) observed the critical genes related to PI3K/Akt and mTOR signaling pathways to be involved in muscle development in grass carp (*Ctenopharyngodonidella*). Therefore, it is of great importance to identify muscle development-related genes and regulative pathways and analyze the molecular mechanisms responsible for fish muscle development.

Recently, with the development of high-throughput sequencing, RNA sequencing (RNA-seq) has provided a rapid and effective way of whole transcriptome analysis to characterize the differentially expressed genes (DEGs) and pathways related to muscle development of fish (Lu et al. 2020; Mohindra et al. 2022; Li et al. 2024a; Luo et al. 2024). For example, in Chinese longsnout catfish (*Leiocassislongirostris*) muscle tissue, 580 DEGs were identified in fish with different growth rates through RNA-seq. And some genes related to feeding behavior (pyruvate kinase and fatty acid-binding protein) were speculated to be the key genes in regulating muscle growth (Li et al. 2024b). In a study on rice flower carp (*Cyprinuscarpio*),403 DEGs were identified in muscle tissue from different-sized individuals through RNA-seq. And some genes involved in promoting muscle contraction (such as gamma-actin, cytoplasmic beta actin, calcium/calmodulin-dependent kinase 2a), and the ubiquitin-proteasome pathway were thought to be related to fish growth (Li et al. 2022). These results indicated that RNA-seq could be an effective tool to identify the candidate genes related to fish growth.

*Cynoglossussemilaevis* belongs to the Cynoglossidae family and is an important economic marine fish species (Meng et al. 2018). Similar to other flatfishes, *C.semilaevis* also has a flat and asymmetrical body axis, and the muscle thickness on the ‘eyed’ side is greater than on the ‘blind’ side. So it is an ideal species for studying muscle development. However, previous studies have mainly focused on muscle development differences in females and males of *C.semilaevis* (Wang et al. 2021; Shi et al. 2023; Wang et al. 2023; Mai et al. 2024); muscle development on the different sides (‘eyed’ side and ‘blind’ side) of the one individual has not been reported. Here, we selected the eyed and blind side muscles in *C.semilaevis* (from the same individual) for muscle tissue structure and comparative transcriptome analysis. The results will not only help to provide potential candidate genes for muscle development but also reveal the regulatory mechanism of the asymmetrical body axis of *C.semilaevis*.

## ﻿Materials and methods

### ﻿Sample collection and preparation

*Cynoglossussemilaevis* used in this study were obtained from a commercial fish farm in Daishan (Zhoushan, China). Five fish (at 2.5 months post-fertilization (mpf), length 5–6 cm) were randomly chosen for this study. All fish sampled were anesthetized with tricaine methanesulfonate (MS-222, 150 mg/L) for subsequent sampling.

The muscle tissue pieces (length 1.5 cm) were dissected out of fish samples at the level of the anal opening and then fixed in 4% paraformaldehyde (PFA) for morphological observation.

Meanwhile, the eyed-side muscle (ESM) and blind-side muscle (BSM) from the same individual were collected for RNA-seq. All samples were frozen in liquid nitrogen, and stored at –80 °C for analysis.

### ﻿Muscle histological assay and muscle thickness measurement

The fixed muscle tissues were dehydrated through a series of graded ethanol and embedded in paraffin wax. Transverse serial wax sections, 4 μm thick, were cut transversely to the long body axis and stained with hematoxylin-eosin (H&E). Sections were mounted on a glass slide with a cover slip and neutral resin, and photographed under light microscopy with a 40× objective lens (Nikon Ni-U, Tokyo, Japan).

**Figure 1.**
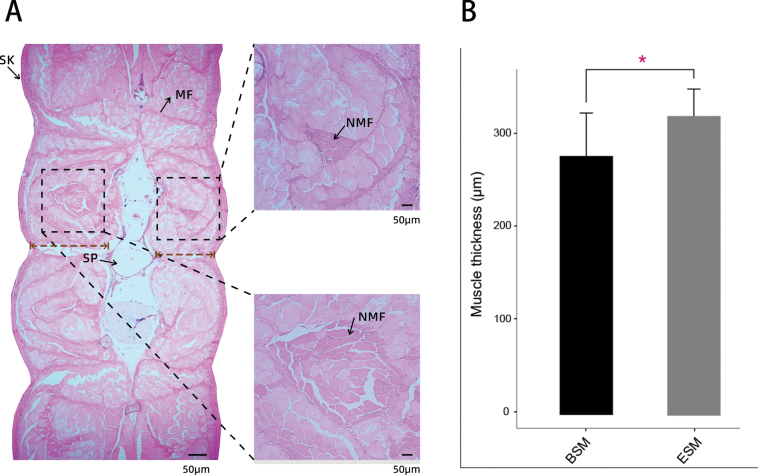
**A** histological transverse section of muscle tissue stained with haematoxylin and eosin in *C.semilaevis*. The arrow demonstrates the position of small new fibers. SK: skin; SC: Spinal cord; N: notochord; P: permysium; scale bar ​= ​50 ​μm **B** the muscle thickness of ESM and BSM. Data was shown as mean ± standard error (SE) (*N* = 5), “*” indicates significant difference (*p* < 0.05).

Three pictures were taken of each sample, and the muscle thickness in the picture was measured. A vertical dotted line was drawn from the spine toward the epidermis as shown in Fig. 1A. The distance was specified as the subscapularis muscle thickness value. The muscle thickness from two sides was analyzed in all samples. Morphological data of muscle thickness was shown by the mean of three pictures.

### ﻿RNA extraction, cDNA library construction, and sequencing

Total RNA was extracted using the TRIzol Reagent kit (Invitrogen, Carlsbad, CA, USA) according to the manufacturer’s protocols (Rio et al. 2010). RNA quality and integrity were evaluated using an Agilent 2100 Bioanalyzer (Agilent Technologies, CA, USA). Then, total RNA was subjected to next-generation sequencing using the DNBSEQ-T7 platform at the Novogene Bioinformatics Technology Co., Ltd. (Tianjin, China). The cDNA libraries of three ESM and three BSM groups were sequenced using the DNBSEQ-T7 platform with 150 bp paired-end reads.

### ﻿Data processing and bioinformatics analysis

Raw sequencing reads of six transcriptome datasets were cleaned by removing the adaptors and low-quality reads using Trimmomatic (v. 0.36) (Bolger et al. 2014). The obtained clean data were then quality-controlled by calculating the values of GC-content, Q20, and Q30 values. After quality control of each sample, the clean data were aligned to the reference gene of *C.semilaevis* (https://www.ncbi.nlm.nih.gov/datasets/genome/GCF_000523025.1/) using HISAT2 (v. 2.0.5) (Mortazavi et al. 2008)

### ﻿Analysis of DEGs

The gene expression levels were calculated by fragments per kilobase of transcript per million reads (FPKMs) mapped (Mortazavi et al. 2008). Differentially expressed genes (DEGs) were identified utilizing the DESeq2 R package, with a significance *P*- value ≤ 0.05 and |log2 (FoldChange)| ≥ 1 (Love et al. 2014). Furthermore, the R package was utilized to perform the analysis and visualization of volcanic diagrams and heat maps of DEGs. Next, these identified DEGs were subjected to functional enrichment analysis, including gene ontology (GO) enrichment analysis and Kyoto Encyclopedia of Genes and Genomes (KEGG) pathway analyses via the R package clusterProﬁler (v. 3.8.1) (Yu et al. 2012). GO terms and KEGG pathways with a *P* ≤ 0.05 were considered signiﬁcantly enriched.

### ﻿Analysis of DAS

Alternative Splicing (AS) was conducted using rMATs (v. 4.1.0) software, including SE, MXE, A5SS, A3SS, and RI for each sample (Shen et al. 2014). For each AS event, rMATS calculated the percentage of exon inclusion (IncLevel) for each sample across the biological triplicates and detected differential IncLevel (IncLevel Difference) between the two groups. The differential alternative splicing (DAS) events were screened and categorized using summary.py in rMATS based on an IncLevelDifference absolute value greater than 0.1 and false discovery rate (FDR)<0.05.

### ﻿Data validation by real-time PCR

To evaluate the reliability of the RNA-seq data, 9 DEGs were randomly chosen for RT-qPCR. 18S rRNA was chosen as the internal control. Total RNA was extracted as described above. The cDNA was synthesized from 1 μg of total RNA using an M-MLV reverse transcriptase (RNase H*^−^*) (TaKaRa Bio Inc., Japan). Primers were designed using Primer 5.0 software (Suppl. material 1). RT-qPCR experiments in a total volume of 12.5 μ contained 0.6 μL of cDNA, 0.25 μL of each primer, 6.25 μL of SYBR Green mix, and 5.15 μL of ddH_2_O. The amplification program was 95 °C for 2 min, 40 cycles at 95 °C for 5 s, 60 °C for 30 s, 72 °C for 3s. The expression level of genes was calculated using the 2^− ΔΔCt^ method, and each sample was performed in triplicate (Livak and Schmittgen 2001).

### ﻿Statistical analysis

All data are shown as the mean ± standard error (SE). Statistical analysis was performed with SPSS 26.0 (SPSS, USA). One-way analysis of variance (ANOVA) was used to compare if there were differences between samples. Significant difference was obtained at *p* < 0.05, which was indicated by an asterisk (*).

## ﻿Results

### ﻿Muscle structure and thickness in eyed side and blind side of *C.semilaevis*

Transverse sections of *C.semilaevis* are shown in Fig. 1A. The muscle cells were arranged neatly, showed an obvious mosaic appearance, and the cell lines were visible. New muscle cell neogenesis (colored dark in Fig. 1A) is produced in the middle parts of the myotomes, neighboring to the horizontal septum, and extends to the end ventrally, which results in a mosaic appearance. In the same visual field, the number of new muscle cells in the ESM was significantly more than that in the BSM (Fig. 1A). Furthermore, the muscle thickness in the ESM (15.30 mm) was significantly greater than that in the BSM (13.33 mm) (Fig. 1B, *P* < 0.05). These results suggest that the mosaic hyperplastic growth of muscle cells may result in differences in muscle development and growth.

### ﻿Summary statistics of transcriptome sequencing

In this study, we constructed a total of six cDNA libraries from two groups, each replicated three times, with an average of 51.89 and 48.06 Mb raw reads for ESM and BSM, respectively. After quality filtering and assessment, an average of 50.52 million and 46.38 million clean reads were yielded, respectively. The GC percentage ranged from 51.77% to 52.62%, with Q20 > 98.38%, Q30 > 94.53%, and no more than 0.02% error rate (Table 1). In addition, over 94% of the clean reads (94.12% - 94.69%) were mapped to the reference genome of *C.semilaevis*. These results indicated that the sequencing data were reliable and could be used for subsequent analysis (Table 1). All raw data obtained in this study were submitted to the NCBI Sequence Read Archive database under the project (PRJNA1196394).

**Table 1.** Sequencing statistics of six *C.semilaevis* muscle transcriptome sequencing samples.

Sample	Raw reads	Clean reads	Q20 (%)	Q30 (%)	GC (%)	Total mapped	Uniquely mapped
ESM1	48891078	47733506	98.38	95.69	49.8	45036059(94.35%)	42379887(88.78%)
ESM2	49458754	48288312	98.42	95.79	50.1	45664547(94.57%)	42834580(88.71%)
ESM3	57334764	55560808	98.37	95.66	50.22	52295278(94.12%)	48688909(87.63%)
BSM1	50618302	49421064	98.64	96.35	50.13	46563975(94.22%)	43617860(88.26%)
BSM2	47020094	44996010	98.8	96.78	49.8	42352606(94.13%)	39497453(87.78%)
BSM3	46550722	44732880	99.02	97.26	49.86	42358102(94.69%)	39536319(88.38%)

### ﻿Identification and comparative analysis of the DEGs

**Figure 2.**
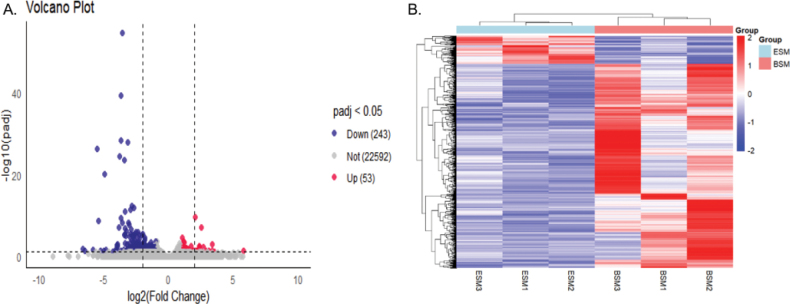
**A** volcano plot of DEGs between ESM and BSM groups (red dots represent up-regulated genes, blue dots represent down-regulated genes, and gray dots represent indistinguishable genes) **B** heatmap plot of DEGs in ESM and BSM groups of *C.semilaevis*. X-axis represents sample name. Y-axis represents the relative expression of DEGs. The different colors indicated changes in the relative expression of DEGs.

In total, 1177 DEGs were identified by comparing the ESM vs BSM group (Suppl. material 1), of which 291 were up-regulated and 886 were downregulated (Fig. 2A). We constructed a clustered heatmap to depict the expression profile of DEGs between ESM vs BSM group. The clustered heatmap showed that the expression patterns within the two groups were mainly divided into main clades, indicating that some DEGs may perform the same or a similar function in a pathway (Fig. 2B).

### ﻿Functional enrichment of DEGs

**Figure 3.**
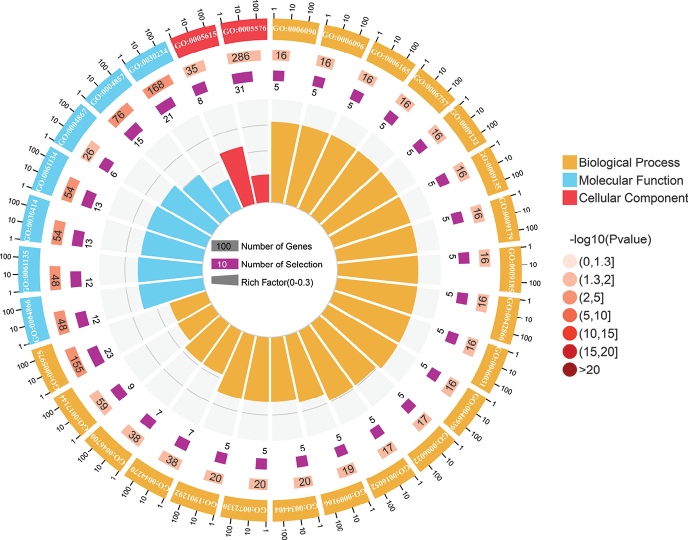
The top most significantly enriched GO terms (top 30) of DEGs between ESM and BSM groups in *C.semilaevis*.

To explore the function of *C.semilaevis* muscle development-related genes and perform the potential functional annotation of DEGs, GO annotation and KEGG enrichment analyses were performed. The GO enrichment analysis showed that 1177 DEGs were enriched in 631 GO terms, and 43 terms were significantly enriched (Suppl. material 2). Fig. 3 shows the top 30 GO terms, of which 21 are associated with biological process (BP), two are related to cellular component (CC), and seven are related to molecular function (MF). These terms mainly affected peptidase inhibitor activity, peptidase regulator activity, carbohydrate metabolic process, endopeptidase inhibitor activity, etc.

**Figure 4.**
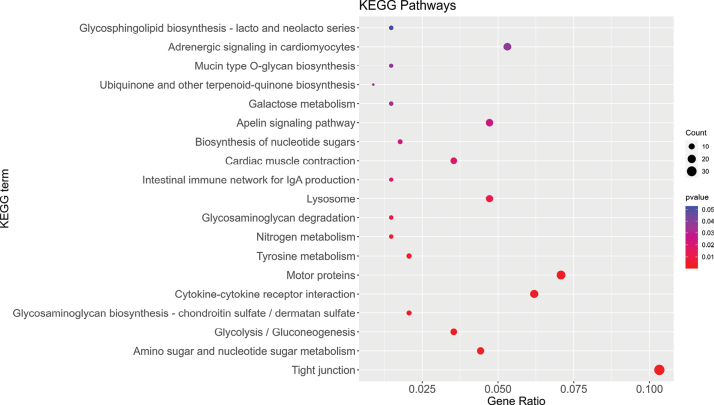
The top 20 enriched KEGG pathways of DEGs between ESM and BSM groups in *C.semilaevis*.

KEGG enrichment analysis indicated that DEGs were annotated into 121 KEGG pathways and 19 pathways were significantly enriched (Suppl. material: Suppl. material 3). As shown in Fig. 4, the main significantly enriched pathways were “cell adhesion molecules”, “Tight junction”, “Amino sugar and nucleotide sugar metabolism”, “ECM-receptor interaction” pathways, etc. Among all the metabolic pathways, glycolysis/gluconeogenesis may be a key regulation pathway in skeletal muscle energy metabolism, including 12 DEGs.

### ﻿Alternative splicing analysis

**Figure 5.**
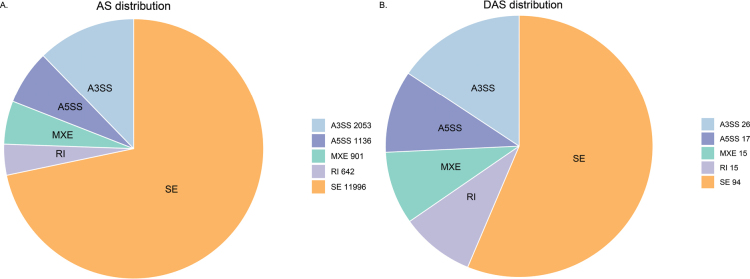
Type distribution of AS events (**A**) and DAS (**B**) in ESM and BSM of *C.semilaevis*.

A total of 16728 AS genes were identified from ESM and BSM groups. The SE, A5SS, A3SS, MXE, and RI splicing types accounted for 71.71%, 6.79%, 12.27%, 5.39%, and 3.84% of all splicing events, respectively (Fig. 5A). After filtering (|IncLevelDifference| > 0.1 and FDR < 0.05), 167 differential AS (DAS) were identified between ESM and BSM. The SE, A5SS, A3SS, MXE, and RI splicing types accounted for 56.29%, 10.18%, 15.57%, 8.98%, and 8.98% of all splicing events, respectively (Fig. 5B).

### ﻿Validation of the DEGs by qRT-PCR

**Figure 6.**
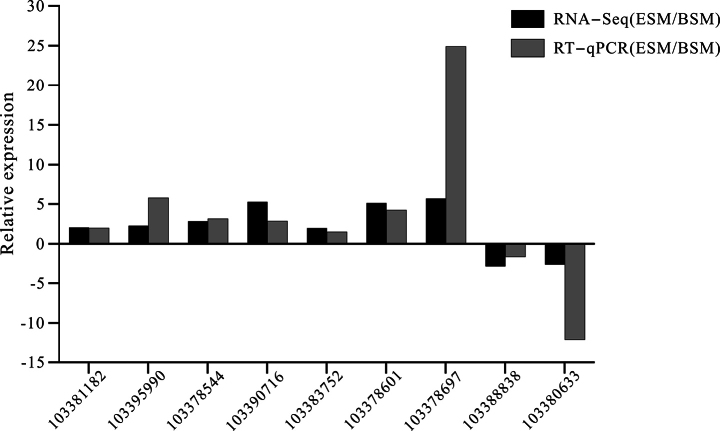
Validation of expression patterns of 9 genes from RNA-seq data by RT-qPCR with 18S gene as an internal control. The values represent the gene expression level of ESM compared with the BSM group.

Nine DEGs were randomly selected for qRT-PCR to verify the reliability of the results from the transcriptome sequencing. As shown in Fig. 6, the relative expression patterns of these genes revealed by qPCR were similar to the RNA-seq results, indicating that the RNA-seq results were reliable.

## ﻿Discussion

### ﻿Muscle morphology of eyed-side muscle and blind-side muscle in *C.semilaevis*

Muscle tissue is the most abundant tissue in the body mass and plays an important role in whole-body metabolism (Zhu et al. 2015). Teleost muscle usually grows in two ways: hypertrophy (increase in fiber size) and hyperplasia (increase in the number of muscle fibers) (Rowlerson and Veggetti 2001). Hyperplasia continues until the fish body grows to a certain stage, after which hypertrophy mainly dominates muscle growth (Johnston et al. 2003). The relative timing of the main hyperplastic growth process concerning the life cycle varies between species (Rowlerson and Veggetti 2001). A previous study found that in the common sole (*Soleasolea*), the first hyperplastic phase starts 12 days post-hatching, and the second hyperplastic phase was already underway at 2.5 months, and by 1 year, the hyperplastic growth of muscle had ceased (Veggetti et al. 1999). In this study, small new fibers were found between the already existing fibers located in the most dorsal and ventral areas of lateral muscle (Fig. 1), which indicated that muscle growth mainly relied on the recruitment of new muscle fibers (hyperplasia) during the juvenile stage of *C.semilaevis*. The same situation was also observed in other species including, European plaice (*Pleuronectesplatessa*) (Brooks and Johnston 1993), turbot (*Scophthalmusmaximus*) (Gibson and Johnston 1995), blackspot seabream (*Pagellusbogaraveo*) (Silva et al. 2009). Additionally, the muscle thickness in the ESM was significantly greater than that of the BSM, which may be caused by the hyperplastic growth of muscle fibers on the eyed side.

### ﻿Transcriptome analysis of ESM and blind-side muscle in *C.semilaevis*

This is the first time that muscle transcriptome has been compared between the eyed side and blind side of *C.semilaevis* using transcriptome sequencing to investigate the potential mechanism of muscle development. In this study, a total of 26,550 unique transcripts were generated and 1177 DEGs were identified (Fig. 2). Among these DEGs, some genes are related to muscle development, for example, myosin family genes and ubiquitin family genes. Myosin family protein is the most abundant and important protein in muscle tissue, which participates in a variety of cellular processes, including cytokinesis, cell polarization, signal transduction, intracellular transport, etc. (Furst et al. 1989; Whiting et al. 1989; Hofmann et al. 2009; Hartman et al. 2011). Studies in salmonids, grass fish, and hybrid grouper indicate that myofibrillar component genes concern growth (Devlin et al. 2009; Sun et al. 2016; Lu et al. 2020). In the present study, several genes related to skeletal muscle myofibrillar components, such as actin, myosin-binding protein C (mybp-C), titin (ttn), troponin (tn), tnnil, myoglobin (mb), and astrotactin-2 (astn-2) were highly expressed in the ESM group. Elevated expression levels of these genes were also found in larger rainbow trout (Salem et al. 2012; Kocmarek et al. 2014). Tn, which is related to the calcium signaling pathway in muscle cells, was up-regulated in the BSM, suggesting that this gene controlling calcium homeostasis also regulates muscle growth in fish (Baar and Esser 1999). It’s identical to the results in grass fish, which promote an increase in muscle tissue (Lu et al. 2020). In addition, mb was also found to be significantly up-regulated in ESM, which indicated that myoglobin directly facilitates aerobic exercise along with the growth of muscle through transporting the oxygen consumed during the process of respiration in muscle cell mitochondria (Wittenberg et al. 1975). However, we also observed up-regulation for different copies of myofibrillar components genes such as myosin light chain 4 (mlc4), myosin regulatory light chain 2 (myl2), troponin C (cTnC), alpha- actin (acta), troponin T-2C (tnt-2C) in ESM group, which were found in other fish (Kocmarek et al. 2014).

In addition, genes involved in the cell cycle (such as murine double minute 2 and cyclin A2) were also significantly up-regulated in the ESM. The organs’ growth depends on an increase both in biomass of individual cells and cell number (Sablowski and Carnier Dornelas 2014). Usually, the increase in cell number is accomplished by cell division (cell cycle progression), which plays an important role in organism development (Goranov et al. 2009). Cyclins are important regulatory proteins, and which bind to cyclin-dependent kinases (CDKs) to regulate the cell cycle progression (Darzynkiewicz et al. 1996). In different animals, cyclin has been demonstrated to play a role in the cellular context of terminally differentiated muscle (Rao and Kohtz 1995). In this study, cyclin A2 was significantly up-regulated in ESM. Li et al. (2024a) showed that cyclin was involved in Yangtze sturgeon (*Acipenserdabryanus*) growth difference as a potential key hub gene through WGCNA analysis. Therefore, the results suggested that the upregulation of genes related to cell cycle might be another endogenous factor that is conducive to different muscle thicknesses between the eyed-side and blind-side muscle in *C.semilaevis*.

Muscle growth reflects the balance between protein synthesis and degradation. The ubiquitin Proteasome Pathway (UPP) is the main system for degrading unnecessary or damaged proteins (Lecker et al. 2006), and the E3-ubiquitin ligases are an important component of this system, which conjugate ubiquitin to the target protein, and then induce protein degradation (David et al. 2011). Furthermore, a large number of ubiquitin system genes is proven to be regulated in atrophying muscles (Lecker et al. 2006). In our study, 22 ubiquitin family genes were identified to be DEGs, 17 genes of which were up-regulated in BSM and 5 were up-regulated in ESM (Suppl. material 1). These results are in accordance with the finding that E3-ubiquitin ligases activate myogenesis in zebrafish. This suggests that ubiquitin system genes could affect muscle development through the ubiquitin-proteasome pathway to promote the degradation of protein in *C.semilaevis*.

Glycolysis is an important metabolic pathway in which glucose is converted into pyruvate producing ATP and NADH for cellular metabolic activity (Hers 1983; Muirhead and Watson 1992). This pathway produces some important precursor metabolites, and also plays a vital role in muscle tissue (Wang et al. 2021). Several studies have shown that glycolysis promotes muscle growth in zebrafish, pigs, and chickens (Lametsch et al. 2006; Teltathum and Mekchay 2009; Wang et al. 2021). Regarding *C.semilaevis*, a previous study indicated that glycolysis/gluconeogenesis contributed to flatfish size dimorphism (Wang et al. 2021). In our study, two important genes in glycolysis (acetyl-CoA synthetase and glucose 6-phosphatase) were more highly expressed in eyed-side muscle tissue than in blind-side muscle tissue. These two DEGs, acs and g6pase, are the key enzymes playing important roles in the regulation of glycolytic/gluconeogenic pathways (Werve et al. 2011; James et al. 2016). The high expression levels of acs and g6pase in the ESM may contribute to the greater muscle thickness in the eyed side of *C.semilaevis*. However, the precise molecular mechanisms of glycolysis/gluconeogenesis and how they participate in muscle development in *C.semilaevis* require further study by the functional analysis of DEGs in this pathway.

## ﻿Conclusions

In the present study, we found the difference in muscle thickness between the eyed side and blind side might be caused by the hyperplastic growth through histological assays. In addition, some DEGs related to muscle development (actin, myopic, titin, tn, tnnil, astn-2, Mdm2, and Ccna2) and the glycolysis/gluconeogenesis pathway were identified and proved to be involved in the muscle development of *C.semilaevis* through comparative transcriptome analysis. This study provides insights into the mechanism regulating muscle development in fish.

## ﻿Additional information

### Author ORCIDs

Jing Yu https://orcid.org/0009-0004-4611-6102

### Data availability

All of the data that support the findings of this study are available in the main text or Supplementary Information.

## Supplementary materials

Supplementary material 1 Primer sequences used for RT-qPCR Data type xlsx https://binary.pensoft.net/file/1271754This dataset is made available under the Open Database License (http://opendatacommons.org/licenses/odbl/1.0/). The Open Database License (ODbL) is a license agreement intended to allow users to freely share, modify, and use this Dataset while maintaining this same freedom for others, provided that the original source and author(s) are credited.Zhenming Lü, Yuzhen Wang, Jing Yu, Yijing Yang, An Xu, Li Gong, Jing Liu, Fenghui Li, Liqin Liu

Supplementary material 2 The catalogue of DEGs in in ESM and BSM groups of *C.semilaevis* Data type xlsx https://binary.pensoft.net/file/1271755This dataset is made available under the Open Database License (http://opendatacommons.org/licenses/odbl/1.0/). The Open Database License (ODbL) is a license agreement intended to allow users to freely share, modify, and use this Dataset while maintaining this same freedom for others, provided that the original source and author(s) are credited.Zhenming Lü, Yuzhen Wang, Jing Yu, Yijing Yang, An Xu, Li Gong, Jing Liu, Fenghui Li, Liqin Liu

Supplementary material 3 The list of enriched GO annotations Data type xls https://binary.pensoft.net/file/1271756This dataset is made available under the Open Database License (http://opendatacommons.org/licenses/odbl/1.0/). The Open Database License (ODbL) is a license agreement intended to allow users to freely share, modify, and use this Dataset while maintaining this same freedom for others, provided that the original source and author(s) are credited.Zhenming Lü, Yuzhen Wang, Jing Yu, Yijing Yang, An Xu, Li Gong, Jing Liu, Fenghui Li, Liqin Liu

Supplementary material 4 The list of enriched KEGG pathways Data type xls https://binary.pensoft.net/file/1271757This dataset is made available under the Open Database License (http://opendatacommons.org/licenses/odbl/1.0/). The Open Database License (ODbL) is a license agreement intended to allow users to freely share, modify, and use this Dataset while maintaining this same freedom for others, provided that the original source and author(s) are credited.Zhenming Lü, Yuzhen Wang, Jing Yu, Yijing Yang, An Xu, Li Gong, Jing Liu, Fenghui Li, Liqin Liu
